# Cost-effectiveness of Transforaminal epidural steroid injections for patients with ACUTE sciatica: a randomized controlled trial

**DOI:** 10.1186/s12891-024-07366-5

**Published:** 2024-04-01

**Authors:** Bastiaan C. ter Meulen, Esther T. Maas, Rien van der Vegt, Johan Haumann, Henry C. Weinstein, Raymond W. J. G. Ostelo, Johanna M. van Dongen

**Affiliations:** 1 Department of Neurology at OLVG Teaching Hospital, Amsterdam, The Netherlands; 2 Department of Epidemiology and Data Sciences, Amsterdam UMC, Vrije Universiteit, Amsterdam Movement Sciences Research Institute Musculoskeletal Health, De Boelelaan 1089a, 1081 HV Amsterdam, The Netherlands; 3 Department of Health Sciences, Faculty of Science, Vrije Universiteit Amsterdam and the Amsterdam Movement Sciences Research Institute, De Boelelaan 1085, 1081 HV Amsterdam, The Netherlands; 4 Department of Pain Medicine and Anesthesiology Zaans MC, Zaandam, The Netherlands; 5 Department of Pain Medicine and Anesthesiology, OLVG, Amsterdam, The Netherlands

**Keywords:** Sciatica, Lumbar disc herniation, Transforaminal epidural steroid injections, Economic evaluation, Randomized controlled trial

## Abstract

**Background:**

Transforaminal epidural injections with steroids (TESI) are increasingly being used in patients sciatica. The STAR (steroids against radiculopathy)-trial aimed to evaluate the (cost-) effectiveness of TESI in patients with acute sciatica (< 8 weeks). This article contains the economic evaluation of the STAR-trial.

**Methods:**

Participants were randomized to one of three study arms: Usual Care (UC), that is oral pain medication with or without physiotherapy, *n* = 45); intervention group 1: UC and transforaminal epidural steroid injection (TESI) 1 ml of 0.5% Levobupivacaine and 1 ml of 40 mg/ml Methylprednisolone and intervention group 2: UC and transforaminal epidural injection (TEI) with 1 ml of 0,5% Levobupivacaine and 1 ml of 0.9% NaCl (*n* = 50). The primary effect measure was health-related quality of life. Secondary outcomes were pain, functioning, and recovery. Costs were measured from a societal perspective, meaning that all costs were included, irrespective of who paid or benefited. Missing data were imputed using multiple imputation, and bootstrapping was used to estimate statistical uncertainty.

**Results:**

None of the between-group differences in effects were statistically significant for any of the outcomes (QALY, back pain, leg pain, functioning, and global perceived effect) at the 26-weeks follow-up. The adjusted mean difference in total societal costs was €1718 (95% confidence interval [CI]: − 3020 to 6052) for comparison 1 (intervention group 1 versus usual care), €1640 (95%CI: − 3354 to 6106) for comparison 2 (intervention group 1 versus intervention group 2), and €770 (95%CI: − 3758 to 5702) for comparison 3 (intervention group 2 versus usual care). Except for the intervention costs, none of the aggregate and disaggregate cost differences were statistically significant. The maximum probability of all interventions being cost-effective compared to the control was low (< 0.7) for all effect measures.

**Conclusion:**

These results suggest that adding TESI (or TEI) to usual care is not cost-effective compared to usual care in patients with acute sciatica (< 8 weeks) from a societal perspective in a Dutch healthcare setting.

**Trial registration:**

Dutch National trial register: NTR4457 (March, 6th, 2014).

**Supplementary Information:**

The online version contains supplementary material available at 10.1186/s12891-024-07366-5.

## Background

Sciatica is characterized by pain radiating to the leg following one of the lumbosacral nerve roots [1]. Other than pain, patients may also experience sensory symptoms and/or weakness of the involved myotome. Approximately 85% of sciatica cases are caused by mechanical compression of the nerve root by a herniated intervertebral disc [2]. The annual incidence of lumbosacral radicular syndrome in the Netherlands has been estimated at 9 per 1000 patient-years [3] and the annual prevalence has been estimated at 17.2 per 1000 patient-years [3]. The prognosis of sciatica is generally described as favourable: within 3 months, 75% of patients are expected to reach bearable pain levels and can resume their work without surgery [4, 5]. Nonetheless, a UK-based study of patients seeking primary care for back-related leg pain, including lumbosacral radicular syndrome, showed that only 55% of patients with lumbosacral radicular syndrome had more than 30% reduction in disability 1 year after their first visit to primary care [6].

In addition to these patient related problems, sciatica poses a major economic burden. Although there are no recent specific cost data for sciatica, in 2017, the total healthcare cost of lower back pain in general (including sciatica) in the Netherlands was estimated to be 937 million [7]. This equals 1.07% of the total expenditure on health care in the Netherlands. Indirect costs due to absenteeism and lost productivity while being at work (i.e., presenteeism) were not included in this cost estimate; and hence the total societal cost will be even higher. Understanding the cost-effectiveness of different management strategies for sciatica, such as medication, physiotherapy, and transforaminal epidural steroid injections (TESI), is important to prevent high healthcare and socioeconomic costs. This requires formal assessments of the best available evidence on the cost-effectiveness of interventions and, where necessary, undertaking economic studies if there is a lack of good quality evidence [8].

TESI is increasingly used in patients with sciatica [9, 10]. In 2021 we described a survey among 80 neurologists (including residents) and 44 anesthesiologists. The results of this survey showed that 40–60% of neurologists think that TESIs are effective in 40% of injected patients and that 23/44(52%) of anesthesiologists think that TESIs are effective in 60–80% of the injected patients [11]. This seems to contradict the current evidence. Four recent systematic reviews and meta-analyses found that TESI only slightly reduced leg pain and disability compared to placebo at short-term follow-up (4–6 weeks) in patients with sciatica, but not at long-term follow-up (> 3 months) [12–15]. According to the GRADE [16], the quality of evidence is low to moderate. Recently, our research group finalized the STeroids against Radiculopathy (STAR) trial, a pragmatic two-center, randomized clinical trial (RCT) assessing the clinical effectiveness of TESI against sciatica [17]. Our results do not support TESI as a standard treatment for patients with acute sciatica (< 8 weeks). Although it is debated as to whether trial-based economic evaluations should still be performed if positive clinical effects are lacking [18], one should be aware that a lack of statistical differences between therapies does not necessarily mean that they are identical. The International Society for Pharmacoeconomics and Outcomes Research (ISPOR) Cost Effectiveness Analysis Randomized Clinical Trial (CEA-RCT) task force therefore recommends that researchers perform CEA if positive clinical results are lacking [19]. The aim of this study was to evaluate the cost-effectiveness of TESI in patients with acute sciatica who participated in the STAR-trial. Within this trial TESI was compared to a (1) treatment regimen of medication only (usual care) and (2) to transforaminal epidural injection with local anesthetic and saline solution (TEI), from a societal perspective.

## Methods

### Study design

An economic evaluation was conducted alongside the STAR trial [17, 20, 21], an RCT evaluating the effectiveness of TESI in patients with acute sciatica. The RCT was conducted in two Dutch hospitals, the Zaans Medisch Centrum (Zaandam) and OLVG Teaching Hospital (Amsterdam), between January ^13,^ 2016, and October ^24,^ 2019.

The following three groups were compared:

- Usual Care (UC): Oral pain medication with or without physiotherapy
- Intervention group 1: Usual care and TESI of 1 ml of 0.5% Levobupivacaine and 1 ml of 40 mg/ml Methylprednisolone
- Intervention group 2: Usual care and transforaminal epidural injection with 1 ml of 0.5% Levobupivacaine and 1 ml NaCI 0.9%

We analyzed three pairwise comparisons:

- Comparison 1 (main comparison): Intervention group 1 versus Usual Care
- Comparison 2: Intervention group 1 versus Intervention group 2
- Comparison 3 (for completeness): Intervention group 2 versus Usual Care

The RCT was approved by the Medical research Ethics Committees United, Nieuwegein, The Netherlands (registration number NL45805.100.15). The protocol was registered in the Dutch Trial Register number NTR4457 (6/03/2014). The CONSORT statement was followed for reporting [22]. See appendix 1.

### Participants

Eligible patients had sciatic symptoms < 8 weeks and were seen by a neurologist at one of the two study centres upon referral by their general practitioners (GPs). Additional inclusion criteria were as follows: a) age between 18 and 75 years; b) magnetic resonance imaging (MRI)-confirmed disc herniation with nerve root impingement causing clinical symptoms; c) pain experienced on average over the last week rated on a numerical rating scale (NRS)(> 4/10); d) good understanding of the Dutch language; and e) Internet access to complete online questionnaires [21]. The exclusion criteria were a) severe weakness of the legs (Medical Research Council ((MRC) score < 3); b) spinal surgery < 1 year at the symptomatic lumbar level; c) lumbar spinal stenosis or spondylolisthesis as the cause of radicular pain diagnosed by MRI; d) pregnancy; and e) severe comorbidity (e.g., cancer) [21].

### Randomization and blinding

Written informed consent was obtained of all patients before entering the study (see Appendix 2 for consent form). Randomization was performed by the study coordinator (BTM) or by one of the two trial nurses using ALEA® software (NKI-AVL, Netherlands). Alea® generated a random schedule of blocks with a maximum size of six. The participants who received a transforaminal injection were blinded to the type of injection.

### Intervention

The procedure was similar for both intervention arms). Participants were brought to a fluoroscopy room and placed in a prone position on the procedure table. Fluoroscopy was used to localize MRI-confirmed disc herniation. Target identification and needle entry were performed according to international procedures [23]. First, the skin was prepped using chlorhexidine. Second, injections were administered using a 22-gauge 100 mm facet tipped needle (Pajunk RGN™). The correct needle position was confirmed by the injection of 0.5–1.5 cc of Joversol 300 mg/ml contrast material (Optiray™ 300, Mallinckrodt). Once an image was obtained demonstrating contrast material spreading into the epidural space medial to a line connecting the ipsilateral lumbar vertebral pedicles, the injection was performed at the level of the herniated disc. The injections were not repeated.

The study participants in intervention group 1 received 1 ml of 0,5% levobupivacaine, followed by 1 ml of 40 mg/ml methylprednisolone in an opaque syringe. The study participants in intervention group 2 received 1 ml 0,5% levobupivacaine followed by 1 ml of 0.9% NaCl. The total volume of the two injections was the same (2 ml).

After epidural injection, washout of the contrast fluid was observed on an X-ray image. The image was saved. Finally, the needle was removed, and the patient was brought to the recovery area.

### Usual care

Patients in all groups used analgesics registered during the trial using online questionnaires [24]. In the case of kinesiophobia, patients were permitted to visit a physiotherapist. There were no restrictions on the use of analgesics or physiotherapy in any group, and their use was monitored. Pain medication, with or without physiotherapy, was planned at the discretion of the attending physician and according to the patient’s personal needs.

### Effect measures

The primary effect measure was health-related quality of life. At baseline, 3 and 6 weeks, and 3 and 6 months after the start of the intervention, the patients’ health states were assessed using the Euroqol-5 dimensions- 3 levels (EQ-5D-3L) [25]. The EQ-5D-3L comprises the following five dimensions: mobility, self-care, usual activities, pain/discomfort, and anxiety/depression. Each dimension had three levels: no problems, problems, and serious problems. The patient indicated her health state by ticking the box next to the most appropriate statement for each of the five dimensions. Each health state was converted into a utility score using the Dutch tariff [26]. Hence, the utility values estimated in this study were indicative of a Dutch person’s value or desirability of patients’ health states. Values ranged between − 0.33 (0 is equivalent to death; negative values are worse than death), and 1 (full health). The EQ-5D-3L is commonly used in cost-utility analyses and, for that reason, applied in this economic evaluation as well [27].

Secondary outcomes were back and leg pain (average previous week) (measured using a 10-cm numerical rating scale [NRS] [28]), physical functioning (measured using the Dutch version of the Roland-Morris Disability Questionnaire [29, 30]), and global perceived recovery (GPR). The latter was measured on a 7-point Likert scale [31] ranging from ‘completely recovered’ to ‘worse than ever,’ which was dichotomized into success (categories ‘completely’ and ‘much recovered’) and non-success (categories ‘slightly recovered,’ ‘no change, ‘slightly worse’, ‘much worse’ and ‘worse than ever’).

### Costs measures

Resource use was assessed using cost questionnaires administered at three and 6 months. In line with Dutch guidelines, costs were assessed from a societal perspective, meaning that all costs were included, irrespective of who paid or benefited [32, 33]. Intervention costs were estimated using the data acquired from the accounting records of the two participating clinics. Data on other healthcare utilization, informal care, unpaid productivity, and absenteeism due to back pain were collected using 3-monthly self-reported web-based cost questionnaires [20]. Healthcare utilization included primary care (e.g., general practitioner care, physiotherapy, manual therapy, chiropractic care, and exercise therapy), secondary care (e.g., hospitalization, and diagnostic and therapeutic interventions), and the use of prescribed and over-the-counter medications. Healthcare utilization was valued using Dutch standard costs and the prices of professional organizations if standard costs were not available. Medication use was valued using prices derived from http://www.medicijnkosten.nl. Informal care included care by family, friends, and other volunteers and was valued according to the proxy good method using an estimate of the hourly cost of a housekeeper. Absenteeism was measured using the Productivity and Disease Questionnaire (PRODISQ) [34]. Absenteeism was valued using sex-specific price weights in accordance with the friction cost approach (friction period = 12 weeks). Unpaid productivity costs included all hours of volunteer work and domestic and educational activities that participants were not able to perform owing to their sciatica; these were also valued using the aforementioned proxy method. All costs were converted to 2020 Euros using consumer price indices. Discounting of costs and effects was not necessary because the follow-up period was 6 months.

### Sample size

Sample sizes were calculated based on a power of 0.9 and a two-sided alpha of 0.05 [20, 21]. These calculations indicated that 48 patients were needed per arm to detect a clinically relevant between-group MD of two points on the 0–10 NRS for leg and back pain (SD = 30). For physical functioning, 22 patients were needed per arm to detect a clinically relevant between-group MD of four points (SD = 4). For dichotomized GPR, 79 patients were needed per arm to detect a clinically relevant between-group difference of 20% [35, 36]. Anticipating a 10% loss to follow-up, 264 patients were included (*n* = 88 per arm).

In accordance with the guidelines of the ‘European Medicines Agency,’ we will only consider one intervention effective over another if statistically significant and clinically relevant differences are found between them for all co-primary outcomes [37].

### Cost-effectiveness and utility analyses

A cost-effectiveness analysis (CEA) and a cost-utility analysis (CUA) were conducted. In the CEA, total costs were related to improvements in back pain, leg pain, functioning, and global perceived effect. In the cost-utility analyses (CUA), total costs were related to the QALYs gained during follow-up. All analyses were performed using intention-to-treat. Baseline characteristics were compared between the intervention and control groups. Missing data for the economic evaluation were handled using multivariate imputation with chained equations. The imputation model included all available cost and effect measure values as well as variables differing between groups at baseline, variables related to the ‘missingness’ of data, and variables related to the outcomes. Ten complete datasets were created so that the loss of efficiency would be less than 5% [18].

The mean between-group cost differences were calculated for total and disaggregated costs (intervention costs, healthcare costs, informal care costs, absenteeism costs, presenteeism costs, and unpaid productivity costs). To determine the mean incremental difference in the cost and effect between the intervention and control groups, we used seemingly unrelated regression (SUR). SUR runs two regressions to determine incremental cost and incremental effect differences simultaneously, adjusting for any potential correlation between costs and effects. The regression for determining the incremental cost difference was adjusted for baseline values (if available) and confounding variables (age, sex, body mass index [BMI], severity of back and leg pain at baseline, and work status).

Incremental cost-effectiveness ratios (ICERs) were calculated by dividing the difference in total costs by the difference in QALYs adjusted for confounders. Uncertainty surrounding the cost differences and ICERs was estimated using bias-corrected and accelerated (BCA) bootstrapping (5000 replications) and graphically presented in cost-effectiveness planes and cost-effectiveness acceptability curves (CEACs). The latter indicates the probability of an intervention condition being cost-effective compared with the control condition at different values of willingness to pay (further referred to as the ceiling ratio). In these analyses, SUR analyses and BCA bootstrapping were nested in multiple imputations, meaning that multiple imputations were used to generate 10 complete datasets, after which the SUR and BCA bootstrapping methods were applied to each of the complete datasets [20, 38]. The intermediate results per completed dataset were pooled using Rubin’s rules [39].

Economic evaluations were performed using STATA (V16) (Stata Corp., College Station, TX, USA).

### Sensitivity analysis

A predetermined sensitivity analysis (SA) was performed to assess the robustness of the results by comparing the friction cost approach with the human capital approach (SA1). Furthermore, complete-case analysis (SA2) and sensitivity analysis using the healthcare perspective (SA3) were performed.

## Results

### Study participants

**Fig. 1:**
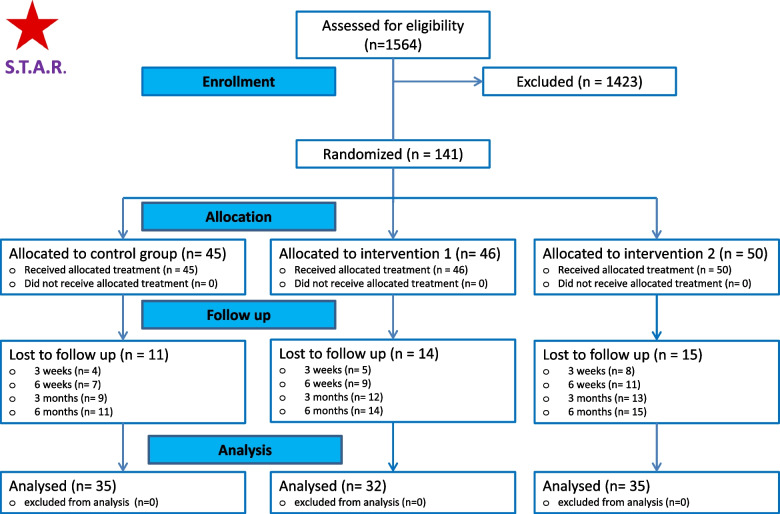
STAR-trial: enrolment, randomization and follow-up

During the study period, 1564 (922 in Amsterdam and 642 in Zaandam) adults with sciatica (regardless of duration) and nerve root compression on MRI were observed. Of these, 141 patients had acute sciatica and were willing to participate in the study. After providing informed consent, 45 patients were assigned to the control group, 46 to intervention group-1, and 50 to intervention group-2. The baseline characteristics are presented in Table 1 and were comparable between the groups. Complete data on all measurements was obtained from 35 (of 45) in the control group, 32 (of 46) in intervention-group 1 (‘steroids’) and 35 (of 50) in intervention-group 2 (‘anesthetic only’). Figure 1 provides an overview of the randomisation between the three study groups and the follow up of participants during 6 months follow-up. All participants were included in the final analysis.

**Table 1:** Baseline variables of included patients

	Control group – Usual care (*n* = 45)	Intervention group 1 Usual care + TESI (*n* = 46)	Intervention group 2 Usual care + TEI (*n* = 50)
**Participant’s characteristics**			
Female- no. (%)	19 (42.2)	26 (56.5)	25 (50.0)
Age- years (SD)	49.2 (12.5)	45.7 (12.9)	48.4 (13.8)
BMI (SD)	26.2 (4.5)	26.4 (5.0)	27.3 (5.6)
Vascular risk factors- no. (%)*	11 (24.4)	13(28.2)	16(32.0)
Education level^a^- no. (%)			
- low	9 (20.0)	9 (19.6)	13 (26.0)
- moderate	24 (53.3)	23 (50.0)	25 (50.0)
- high	12(26.7)	14 (30.4)	12 (24.0)
Married or with a partner- no. (%)	35 (77.8)	28 (60.9)	36 (72.0)
Having a paid job- no. (%)	41 (91.1)	43 (93.5)	45 (90.0)
Primary outcomes*			
Leg pain intensity score^b^ - mean(SD)	7.3 (2.0)	7.8 (1.8)	7.7 (1.9)
Back pain intensity score^b^- mean (SD)	5.3 (3.1)	5.9 (2.7)	5.8 (3.0)
Physical functioning^c^- mean (SD)	17.5 (3.1)	18.2 (4.2)	16.6 (4.6)
Secondary outcomes*			
Health-related quality of life^d^- mean (SD)	0.74 (0.07)	0.71 (0.07)	0.73 (0.07)

*Usual care* Oral medication: *TESI* Transforaminal Epidural Steroid Injection: *TEI* Transforaminal Epidural Injection

*n = 136

^a^Low includes preschool, primary school, or lower secondary school; moderate includes higher secondary school or undergraduate; high includes tertiary, university, or postgraduate

^b^Leg pain and back pain intensity were measured by means of the numerical pain rating scale (NRS), whereby patients were asked to measure their average pain over the previous 24 hours on a 0–10 scale, with 0 indicating no pain and 10 indicating the worst imaginable pain

^c^The extent of physical functioning was measured on the Roland Disability Scale of Sciatica (scores ranging from 0 to 23, with higher scores indicating greater physical functioning)

^d^Health-related quality of life was assessed using the Euroqol 5- dimensions - 3 levels (EQ-5D-3L) and converted to utility values ranging from 0 (equal to death) to 1 (equal to full health) using the Dutch tariff

### Effects

None of the between-group differences in effects were statistically significant, for any of the outcomes (QALY, back pain, leg pain, functioning, and global perceived recovery) at the 26-weeks follow-up [17].

### Costs

The mean cost per patient in the various study groups and the unadjusted mean cost differences between the groups are shown in Table 2. For both intervention groups, the cost per participant was estimated to be €486 per participant. The total societal costs were €21,724 (SEM 2461) per participant for intervention group 1, €21,337 (SEM2087) per participant for intervention group 2, and €21,400 (SEM 2165) for the usual care group.

**Table 2:** Mean cost per patient in the various study groups, and unadjusted mean cost differences between groups

Cost category	Intervention group 1 – Usual care + TESI Mean (SEM)	Intervention group 2 – Usual care + TEI Mean (SEM)	Control group – Usual care Mean (SEM)	Comparison 1 (Int. group 1 vs. Control)		Comparison 2 (Int. group 1 vs. Int. group 2)		Comparison 3 (Int. group 2 vs. Control)	
				Unadjusted	Adjusted*	Unadjusted	Adjusted*	Unadjusted	Adjusted*
Intervention costs	486 (1)	486 (1)	0 (0)	486 (485 to 487)	486 (485 to 487)	0 (−2 to 1)	0 (−1 to 2)	486 (485 to 487)	486 (485 to 487)
Other healthcare costs	2081 (269)	2394 (722)	2448 (354)	−367 (− 1301 to 401)	− 690 (− 1816 to 165)	− 313 (− 2719 to 686)	− 233 (− 2442 to 751)	−54 (− 1126 to 2271)	−72 (− 1257 to 2636)
*Prim. healthcare costs*	*1179 (238)*	*1018 (233)*	*1588 (299)*	*− 409 (− 1162 to 164)*	*−650 (− 1622 to 5)*	*161 (− 356 to 707)*	*218 (−264 to 745)*	*−570 (− 1292 to 9)*	*− 635 (− 1396 to − 3)*
*Sec. healthcare costs*	*817 (170)*	*1281 (640)*	*761 (170)*	*57 (− 373 to 479)*	*−22 (− 502 to 423)*	*− 463 (− 2992 to 312)*	*−440 (− 2781 to 317)*	*520 (− 258 to 2978)*	*566 (− 303 to 3328)*
*Medication costs*	*86 (15)*	*95 (22)*	*99 (12)*	*−14 (− 43 to 27)*	*− 19 (− 50 to 25)*	*− 10 (− 69 to 34)*	*−10 (− 68 to 30)*	*−4 (− 40 to 60)*	*−3 (− 41 to 65)*
Informal care costs	1669 (608)	1563 (588)	2031 (610)	− 362 (− 1785 to 959)	− 569 (− 1992 to 931)	105 (− 1594 to 1302)	61 (− 1879 to 1226)	− 468 (− 1885 to 1229)	−322 (− 1635 to 1333)
Absenteeism costs	12,959 (2144)	13,153 (1646)	12,090 (1972)	869 (− 3715 to 5099)	2410 (− 2116 to 6350)	− 194 (− 4636 to 4126)	684 (− 3176 to 4368)	1063 (− 3415 to 5432)	1833 (− 2151 to 5498)
Presenteeism costs	3288 (759)	2788 (544)	3374 (613)	−86 (− 1649 to 1428)	333 (− 1131 to 1806)	499 (− 941 to 1866)	858 (− 455 to 2053)	−585 (− 2080 to 900)	− 631 (− 2065 to 818)
Unpaid productivity costs	1242 (391)	952 (284)	1458 (289)	−216 (− 956 to 574)	− 251 (− 1058 to 734)	289 (− 422 to 1019)	269 (− 484 to 1148)	− 505 (− 1206 to 213)	−524 (− 1271 to 184)
**Total societal costs**	**21,724 (2461)**	**21,337 (2087)**	**21,400 (2165)**	**323 (− 4806 to 5117)**	**1718 (− 3020 to 6052)**	**387 (− 5045 to 5631)**	**1640 (− 3354 to 6106)**	**−64 (− 5325 to 5441)**	**770 (− 3758 to 5702)**

*Usual care* Oral medication: *TESI* Transforaminal Epidural Steroid Injection: *TEI* Transforaminal Epidural Injection

*Comparisons were adjusted for center, gender, level of herniated disc, loss of strength, loss of feel, BMI, age

Total values are depicted in bold font

*SEM* standard error of the mean

The adjusted mean difference in total societal costs was €1718 (95% confidence interval [CI], − 3020 to 6052) for comparison 1 (intervention group 1 versus usual care), €1640 (95%CI: − 3354 to 6106) for comparison 2 (intervention group 1 versus intervention group 2), and €770 (95%CI: − 3758 to 5702) for comparison 3 (intervention group 2 versus usual care). Except for intervention costs, none of the aggregate and disaggregate cost difference was statistically significant.

### Cost-effectiveness

The results of the cost-effectiveness analyses, including differences in costs, differences in effects, ICERs, and distributions of bootstrapped cost-effect pairs across the four quadrants of the CE plane, can be found in Table 3 for all comparisons.

**Table 3:** Cost-effectiveness analysis results (main analysis)

Outcome	Sample size Outcome			∆C (95%CI)	∆E (95%CI)	ICER	Distribution CE-plane (%)			
**Comparison 1** **(Int. group 1 (Usual care + TESI) vs. Control (Usual care))**										
	**Int. Group 1**	**Control**		**€**	**Points**	**€/point**	**NE**	**SE**	**SW**	**NW**
**QALYs (0–1)**^**1**^	46	45		1839 (− 2891 to 6281)	0.008 (− 0.41 to 0.57)	234,478	44.5	17.1	8.1	30.3
**Back pain (0–10)** ^**2**^	46	45		1694 (− 3037 to 6177)	0.6 (− 0.5 to 1.7)	2742	11.4	3.7	23.2	61.7
**Leg pain (0–10)** ^**3**^	46	45		1730 (− 2886 to 6247)	−0.4 (− 2.2 to 1.4)	− 4409	54.4	16.5	10.2	19.0
**Functioning (0–23)** ^**4**^	46	45		1746 (− 3029 to 6240)	1.5 (− 1.7 to 4.7)	1158	12.0	5.1	21.5	61.4
**Global Perceived Effect** ^**5**^	46	45		1662 (− 2903 to 6156)	0.04 (−0.02 to 0.11)	39,295	71.5	23.5	0.4	4.6
**Comparison 2** **(Int. group 1 (Usual care + TESI) vs. Int. group 2 (Usual care + TEI))**										
	**Int. Group 1**	**Int. Group 2**		**€**	**Points**	**€/point**	**NE**	**SE**	**SW**	**NW**
**QALYs (0–1)**^**1**^	46	50		1692 (− 3245 to 6146)	−0.011 (−0.061 to 0.038)	− 150,243	20.5	11.0	15.9	52.6
**Back pain (0–10)** ^**2**^	46	50		1616 (− 3303 to 6113)	−0.01 (− 1.3 to 1.3)	− 12,3742	37.9	16.1	11.5	34.5
**Leg pain (0–10)** ^**3**^	46	50		1644 (− 3280 to 6062)	−0.1 (−1.5 to 1.3)	− 25,791	40.3	15.5	11.7	32.5
**Functioning (0–23)** ^**4**^	46	50		1644 (− 3463 to 6167)	0.99 (−2.15 to 4.12)	1664	18.6	6.8	20.4	54.2
**Global Perceived Effect** ^**56**^	46	50		–	–	–	–	–	–	–
**Comparison 3** **(Int. group 2 (Usual care + TEI) vs. Control (Usual care)**										
	**Int. Group 2**	**Control**		**€**	**Points**	**€/point**	**NE**	**SE**	**SW**	**NW**
**QALYs (0–1)**^**1**^	50	45		735 (− 3815 to 5734)	0.012 (−0.040 to 0.064)	59,442	41.9	26.0	12.3	19.9
**Back pain (0–10)** ^**2**^	50	45		802 (− 3895 to 5824)	0.8 (−0.4 to 1.9)	1033	7.4	4.1	32.5	56.0
**Leg pain (0–10)** ^**3**^	50	45		770 (− 3893 to 5641)	−0.2 (−1.7 to 1.4)	− 4136	42.4	21.3	15.2	21.0
**Functioning (0–23)** ^**4**^	50	45		772 (− 3778 to 5627)	0.4 (−3.3 to 4.0)	2135	27.0	14.6	22.7	35.7
**Global Perceived Effect** ^**5**^	50	45		747 (− 3863 to 5627)	0.05 (−0.01 to 0.10)	16,564	67.9	32.1	0.0	0.0

*Usual care* Oral medication: *TESI* Transforaminal Epidural Steroid Injection: *TEI* Transforaminal Epidural Injection

*C* Costs: *E* Effects: *ICER* Incremental Cost-Effectiveness Ratio: *CE-plane* Cost-Effectiveness plane: *NE* Northeast-Quadrant: *SE* Southeast-Quadrant: *NW* Northwest-Quadrant: *ZW* Southwest-Quadrant: Cost differences were adjusted for age, gender, body mass index (BMI), and severity of back and leg pain at baseline

^1^adjusted for baseline utility values, work status, age, gender

^2^adjusted for baseline values, level of herniated disc

^3^adjusted for baseline values, age, gender, body mass index (BMI), and severity of back and leg pain at baseline. Lasègue’s sign

^4^adjusted for baseline values, work status, gender

^5^percentage point (difference in percentage recovered between intervention -and control group) adjusted for work status

^6^No patients were recovered, cannot be estimated

At 6 months, the ICER for QALYs was €234,478 for comparison 1 (intervention group 1 versus usual care), indicating that the additional societal cost in intervention group 1 compared to usual care was €234,478 per QALY gained. This ICER shows that the intervention was -on average- “more costly” and “more effective” than usual care, which was also the case for leg pain and perceived recovery. ICERs for back pain and physical functioning, on the other hand, showed that the intervention was on average “more costly” and “less effective,” indicating that it was dominated by usual care.

For comparison 2 (intervention group 1 versus intervention group 2), ICERs for QALYs and physical functioning indicated that intervention 1 was dominated intervention 2 for these outcomes (i.e. on average “more costly” and “less effective”), while ICERs for back and leg pain showed that intervention 1 was on average “more costly” and “more effective.” It should be noted that outcomes for self-perceived recovery are lacking because all participants in both groups indicated recovery after 6 months.

For comparison 3 (intervention group 2 versus usual care), ICERs indicated that the intervention was dominated by usual care for back pain and physical functioning (i.e. on average “more costly” and “less effective”), while it was “more costly” and “more effective” for QALYs, leg pain, and self-perceived recovery.

**Fig. 2:**
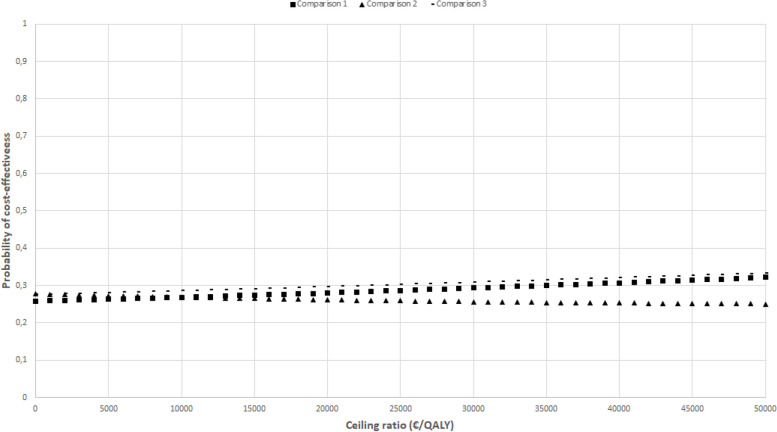
Cost-effectiveness acceptability curve indicating the probability of the intervention conditions’ being cost-effectiveness compared with control for different ceiling ratios (€) for quality-adjusted life-years

**Fig. 3:**
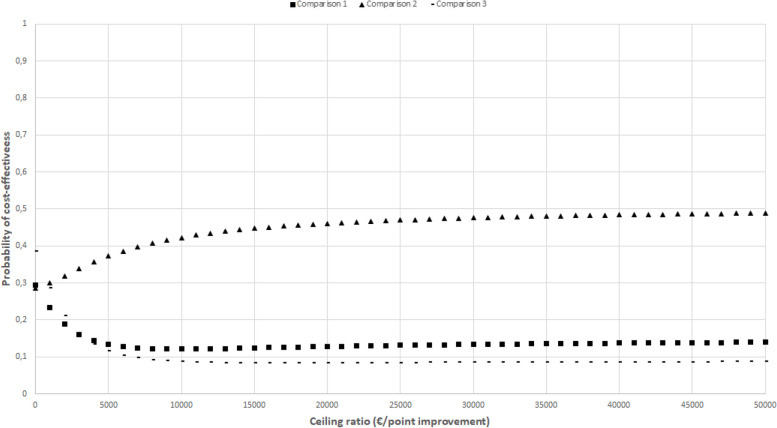
Cost-effectiveness acceptability curve indicating the probability of the intervention conditions’ being cost-effectiveness compared with control for different ceiling ratios (€) for back-pain

**Fig. 4:**
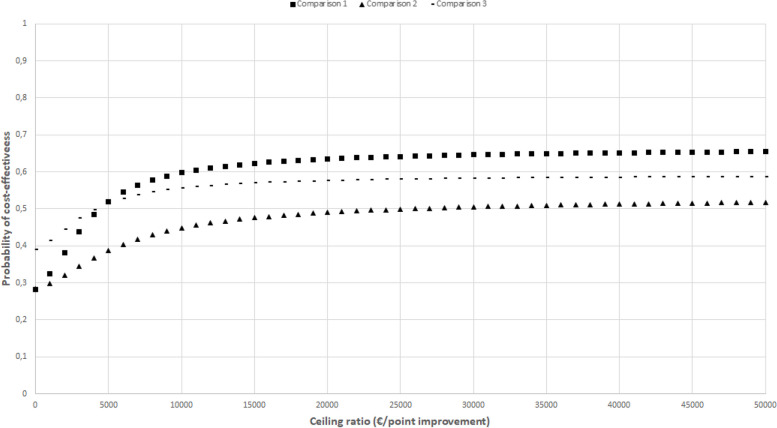
Cost-effectiveness acceptability curve indicating the probability of the intervention conditions’ being cost-effectiveness compared with control for different ceiling ratios (€) for leg pain

**Fig. 5:**
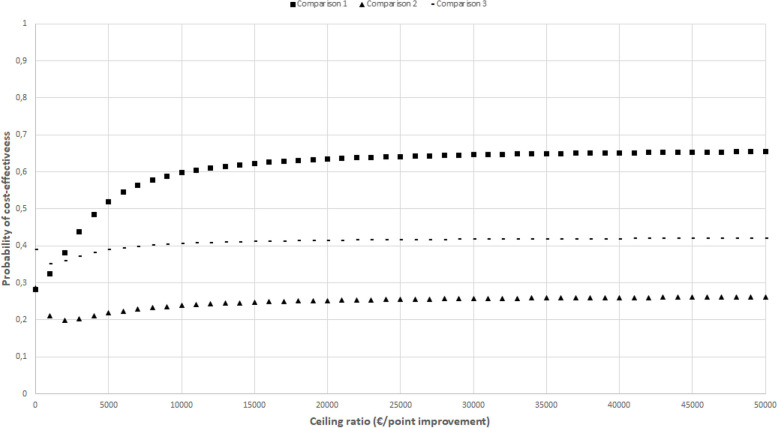
Cost-effectiveness acceptability curve indicating the probability of the intervention conditions’ being cost-effectiveness compared with control for different ceiling ratios (€) for physical functioning

**Fig. 6:**
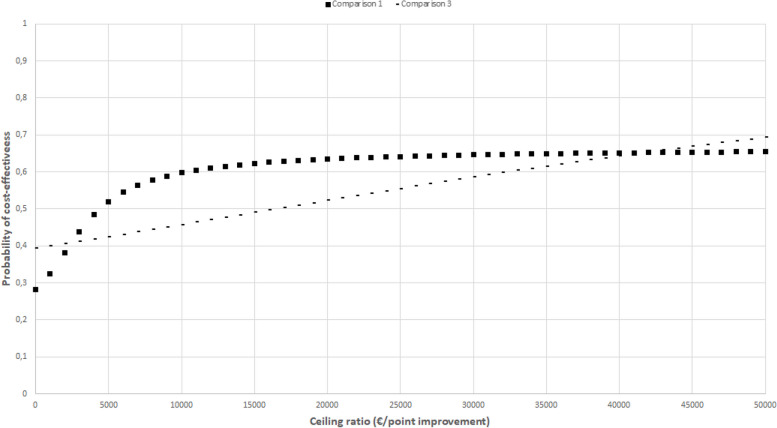
Cost-effectiveness acceptability curve indicating the probability of the intervention conditions’ being cost-effectiveness compared with control for different ceiling ratios (€) for self-perceived recovery

The CEACs in Figs. 2, 3, 4, 5, and 6 show that the maximum probability of both interventions being cost-effective compared to usual care was low (< 0.7) for all effect measures. A probability of cost-effectiveness of 0.7 means that if the intervention is implemented, it will indeed be cost-effective in 70% of cases, whereas in 30% of cases it will not.

### Sensitivity analyses

In line with the main analysis, between-group differences in total costs and effects were not significant in any of the sensitivity analyses (Appendix 3). The complete-case analysis showed a small statistically significant effect on GPR for comparison 1 (0.04 (95%CI 0.02 to 0.07)). However, the overall conclusion of this study does not change when any of the assumptions of the sensitivity analyses are used.

### Adverse effects

No adverse events were observed. None of the participants withdrew from the trial out of safety measures.

## Discussion

### Principal findings

In this study, the cost and cost-effectiveness of adding an invasive treatment, that is, TESI to usual care in the treatment of acute sciatica (< 8 weeks) in the secondary care setting was assessed. The results suggest that adding TESI to usual care is not cost-effective compared with usual care alone in patients with acute sciatica from a societal perspective in a Dutch healthcare setting. That is, the maximum probability for adding invasive treatment TESI of being cost-effective in comparison to the control was low for all possible outcomes (< 0.7) and all comparisons. Comparisons 2 and 3 show comparable results.

Sensitivity analyses confirmed these results, although a small positive effect on GPR was found in the complete-case analysis for comparison 1 (∆E 0.04 (95%CI 0.02–0.07)). This discrepancy with the main analysis is likely caused by the selective dropout of participants, and as multiple imputation was used in the main analysis to handle missing data, we consider these results to be more valid.

All in all, these results suggest that TESI or TEI in additional to usual care (oral pain medication) is not cost-effective compared with usual care alone from a societal perspective in acute sciatica patients in the Dutch healthcare setting.

### Comparison to the literature

*Price* et al [40] conducted a prospective, double-blind randomized trial in the UK assessing the (cost-)effectiveness of TESI versus a placebo injection of normal saline into the interspinous ligament in 228 participants with sciatica during a 12-month follow-up. The most important outcomes were the number needed to treat to realize a 75% improvement in pain relief and functional status at 3 weeks, which was 11.4 (*p* = 0.017), and the cost per QALY for one epidural steroid injection which was £25,746 from a provider’s perspective and £31,904 from a purchaser’s perspective. Given the fact that in this trial there was no clinical benefit of TESI over placebo between 6 and 52 weeks, the authors concluded that TESIs were not cost-effective. However, *Price* et al did not assess the costs of informal care as and productivity losses. In addition *Price* et al assumed that both treatment groups received similar pain medication that did not differ between groups. As a result costs of pain medication were not explicitly measured.

*Spijker* et al [41] performed a pragmatic, randomized, controlled, single-blinded trial in Dutch general practices (*n* = 63) and compared one segmental epidural steroid injection containing 80 mg triamcinolone (intervention) to usual care (taking analgesics as needed, and maintaining normal daily activities as much as possible) during 52 weeks. Mean total costs were €4414 in the intervention group and €5121 in the control group, a difference that was mostly due to differences in lost productivity. The point estimate for the ICER was - €730, meaning that a one point decrease on the NRS back pain scale in one patient during the course of 1 year was associated with a saving of €730 compared with usual care. The cost-effectiveness acceptability curve (CEAC) showed that without additional investments, the probability that epidural corticosteroid injections are cost-effective was more than 80%. *Spijker* et al concluded that the effect on pain and disability of epidural corticosteroid injections in sciatica is small, but significant (contrary to this RCT that found no clinically relevant differences between groups [16, 17]), and at lower costs and recommended that ‘policymakers could consider segmental epidural steroid injections as an additional treatment option’. The difference in results between our trial and that of *Spijker* et al [41] could be explained by differences in the way both studies handled missing data (i.e. multiple imputation in our study versus a complete-case analysis in the study of *Spijker* et al) and baseline imbalances (i.e. regression-adjustment in our study versus no adjustment in the economic evaluation of *Spijker* et al) as well as the absence of informal care, unpaid productivity, and presenteeism costs in the study of *Spijker* et al.

### Strengths and limitations

The major strength of the STAR trial is its pragmatic design, meaning that its set-up resembled real-life routine practice conditions as much as possible [42]. Thus, the STAR trial enabled us to evaluate TESI against acute sciatica under circumstances directly in line with clinical practice, making the current results generalizable to Dutch clinical practice. The current analyses were also conducted using state of the art methods. That is, multiple imputation was used for handling missing data, regression-based adjustment for handling baseline imbalances, non-parametric bootstrapping for handling the skewed nature of cost data, and seemingly unrelated regression for handling the correlation between costs and effects [43]. This is important, as previous research indicates that using less optimal methods may notably impact results and might even impact on the conclusions of trial-based economic evaluations [44]. Another strength is that not only QALYs, functional status, and pain intensity were used as an outcome measure in the economic evaluation, but also perceived recovery as measured by the GPR. The similarity in results from four different outcome measures gave confidence in the robustness of results. Moreover, varies sensitivity analyses were performed that showed the robustness of results as well. All these attributes support the validity of the findings observed in this study.

This trial also has limitations. One limitation is that we used one particular injection technique, i.e. TESI. Other epidural injection techniques, such as caudal epidural approach [45] or echography- guided transforaminal approach [46], might further reduce costs and improve the cost-effectiveness of epidural injections. The latter techniques were not chosen because in The Netherlands there is a strong preference for TESI (usual care within the Pain Department). A second limitation is the use of self-reported retrospective cost questionnaires that may have introduced recall bias and/or “social desirability bias. However, as it seems unlikely that recall bias or the degree to which participants gave socially desirable answers systematically differed between groups, it is not expected that self-report biased the results. A third limitation concerns the missing data. We used multiple imputation (MI) to deal with this limitation. MI is considered a powerful statistical tool, as it accounts for uncertainty by replicating the incomplete dataset multiple times and substituting the missing values with plausible ones [44]. A fourth limitation was the use of the three-level version of the EQ-5D rather than the 5-level version to measure QALYs [47]. This is a possible limitation, as the EQ-5D-3L was found to have significantly higher floor effects for the health dimension pain/discomfort and ceiling effects for the health dimensions mobility, self-care, pain/discomfort, anxiety/depression, compared with the EQ-5D-5L. However, as the floor and ceiling effects are likely to be equal in all study groups, we do not expect our use of the EQ-5D-3L to have severely biased our results. As a fifth limitation, opioid use was analysed on an aggregate level, and no detailed on the type, number, and dosage of opioids that the patients used was collected. When measuring pain, it is important to measure the medication that affects pain. We recommend future studies to measure this on a detailed level.

## Conclusion

Although a common treatment among patients with sciatica due to an MRI-confirmed herniated lumbar disc, evidence from our trial-based economic evaluation suggests that TESI in the acute phase (< 8 weeks) cannot be considered cost-effective compared to usual care from a societal perspective in a Dutch healthcare setting during a 6 months follow up period. Therefore, the current status of this treatment in the Dutch healthcare setting should be reconsidered.

### Supplementary Information

**Additional file 1.** Appendix I CONSORT-statement.

**Additional file 2.** Appendix II Informed consent form.

**Additional file 3.** Appendix III Cost-effectiveness analysis results (Sensitivity analyses).

## Data Availability

The datasets used and/or analysed during the current study are available from the corresponding author on reasonable request.

## Abbreviations

AE: Adversed effect
AR: Adversed reaction
CI: Confidence interval
CEA: Cost Effectiveness Analysis
CEAC: Cost-effectiveness acceptability curve
CUA: Cost-utility analysis
EQ-5D-3L: Euroqol-5 dimensions- 3 levels
EQ-5D-5 L: Euroqol-5 dimensions- 5 levels
FDA: Food and drug administration
GP: General practitioner
GPR: Global perceived recovery
GRADE: Grades of recommendation, assessment, development and evaluation
ICER: Incremental cost-effectiveness ratios
MD: Mean difference
MI: Multiple Imputation
MRC: Medical research council
MRI: Magnetic resonance imaging
NRS: Numerical rating scale
NTR: Nederlands trial register
Prodisc: Productivity and disease questionnaire
Qaly: Quality adjusted life year
RCT: Randomized controlled trial
RDQ: Roland-Morris disability questionnaire
SUR: Seemingly unrelated regression (SUR)
TEI: Transforaminal epidural injection
TESI: Transforaminal epidural injections with steroids
VAS: Visual analogous scale
