# Supplementary material for: Cost-effectiveness of Transforaminal epidural steroid injections for patients with ACUTE sciatica: a randomized controlled trial
Source: BMC Musculoskelet Disord. 2024 Apr 1;25:247. doi: 10.1186/s12891-024-07366-5 (PMC10983727; doi:10.1186/s12891-024-07366-5)
Supplement: Supplementary file 2 — Additional file 2. Appendix II Informed consent form. [file 12891_2024_7366_MOESM2_ESM.docx]

**Appendix II: Epidural corticosteroids in lumbosacral radicular syndrome – Informed consent form**

I read the test subject information letter. I was able to ask additional questions. My questions were answered. I had enough time to decide whether to participate.

I know that participating is voluntary. I know that I can decide at any time not to participate anymore. I don't have to give a reason for that.

I consent to tell my GP that I am participating in this study (if applicable).

I consent to tell the specialist(s) treating me that I am participating in this study (if applicable).

I know that some people can see my data. Those people are listed in the General brochure.

I consent to the use of my data, for the purposes as set out in the information letter.

I consent to the retention of my research data for 15 years after the end of this study.

I want to participate in this study.

Subject's name:

Signature:

Date : __ / __ / __

-----------------------------------------------------------------------------------------------------------------

I hereby declare that I have fully informed this subject about the aforementioned study.

If information becomes known during the study that could affect the subject's consent, I will

inform him/her in a timely manner.

Investigator's name (or representative):

Signature: Date: __ / __ / __

-----------------------------------------------------------------------------------------------------------------

Additional information was provided by (if applicable):

Name:

Function:

Signature: Date: __ / __ / __

-----------------------------------------------------------------------------------------------------------------
