# Supplementary material for: Cost-effectiveness of Transforaminal epidural steroid injections for patients with ACUTE sciatica: a randomized controlled trial
Source: BMC Musculoskelet Disord. 2024 Apr 1;25:247. doi: 10.1186/s12891-024-07366-5 (PMC10983727; doi:10.1186/s12891-024-07366-5)
Supplement: Supplementary file 3 — Additional file 3. Appendix III Cost-effectiveness analysis results (Sensitivity analyses). [file 12891_2024_7366_MOESM3_ESM.docx]

**Appendix III: Cost-effectiveness analysis results (Sensitivity analyses)**

| **HEALTHCARE PERSPECTIVE** | | | | | | | | | |
| --- | --- | --- | --- | --- | --- | --- | --- | --- | --- |
| **Outcome** | **Sample size Outcome** | | **∆C(95%CI)** | **∆E(95%CI)** | **ICER** | **Distribution CE-plane (%)** | | | |
| **Comparison 1**  **(Int. group 1 (Usual care + TESI) vs. Control (Usual care))** | | | | | | | | | |
|  | **Int. Group 1** | **Control** | **€** | **Points** | **€/point** | **NE** | **SE** | **SW** | **NW** |
| **QALYs (0-1)^1^** | 46 | 45  45  45  45  45 | -178 (-1323 to 669) | 0.008 (-0.041 to 0.056) | -20,996 | 18.6 | 44.2 | 19.1 | 18.1 |
| **Back pain (0-10) ^2^** | 46 |  | -191 (-1350 to 650) | 0.6 (-0.5 to 1.7) | -309 | 6.1 | 9.4 | 53.4 | 31.0 |
| **Leg pain (0-10) ^3^** | 46 |  | -225 (-2422 to 745) | -0.4 (-2.2 to 1.4) | 562 | 21.1 | 35.9 | 23.5 | 19.5 |
| **Functioning (0-23) ^4^** | 46 |  | -197 (-1317 to 640) | 1.5 (-1.7 to 4.8) | -131 | 3.5 | 13.2 | 51.4 | 31.9 |
| **Global Perceived Effect ^5^** | 46 |  | -198 (-1345 to 639) | 0.04 (-0.02 to 0.11) | --4,670 | 40.1 | 54.7 | 3.0 | 2.3 |
| \| **Comparison 2**  **(Int. group 1 (Usual care + TESI) vs. Int. group 2 (Usual care + TEI))** \| \| \| \| \| \| \| \| \| \| \| \| --- \| --- \| --- \| --- \| --- \| --- \| --- \| --- \| --- \| --- \| --- \| \|  \| **Int. Group 1** \| **Int. Group 2** \| **€** \| **Points** \| **€/point** \| **NE** \| **SE** \| **SW** \| **NW** \| | | | | | | | | | |
| **QALYs (0-1)^1^** | 46 | 50  50  50  50  50 | -207 (-2315 to 753) | -0.011 (-0.061 to 0.038) | 18,258 | 8.4 | 23.0 | 35.5 | 33.0 |
| **Back pain (0-10) ^2^** | 46 |  | -195 (-2344 to 746) | -0.03 (-1.2 to 1.1) | 6,693 | 19.1 | 32.8 | 24.8 | 23.4 |
| **Leg pain (0-10) ^3^** | 46 |  | -225 (-2422 to 745) | -0.1 (-1.5 to 1.3) | 2,377 | 21.1 | 35.9 | 23.5 | 19.5 |
| **Functioning (0-23) ^4^** | 46 |  | -233 (-2440 to 725) | 0.99 (-2.15 to 4.12) | -211 | 8.3 | 16.0 | 43.5 | 32.2 |
| **Global Perceived Effect ^56^** | 46 |  | - | - | - | - | - | - | - |
| \| **Comparison 3**  **(Int. group 2 (Usual care + TEI) vs. Control (Usual care)** \| \| \| \| \| \| \| \| \| \| \| --- \| --- \| --- \| --- \| --- \| --- \| --- \| --- \| --- \| --- \| \|  \| **Int. Group 2** \| **Control** \| **€** \| **Points** \| **€/point** \| **NE** \| **SE** \| **SW** \| **NW** \| | | | | | | | | | |
| **QALYs (0-1)^1^** | 50 | 45  45  45  45  45 | 385 (-812 to 2985) | 0.012 (-0.040 to 0.064) | 31,468 | 40.9 | 27.5 | 6.7 | 24.9 |
| **Back pain (0-10) ^2^** | 50 |  | 464 (-747 to 3218) | 0.8 (-0.4 to 1.9) | 572 | 6.8 | 4.2 | 27.1 | 61.9 |
| **Leg pain (0-10) ^3^** | 50 |  | 410 (-784 to 3082) | -0.2 (-1.7 to 1.4) | -2,909 | 38.7 | 22.2 | 10.5 | 28.6 |
| **Functioning (0-23) ^4^** | 50 |  | 409 (-778 to 3046) | 0.4 (-3.3 to 4.0) | 1,230 | 24.1 | 17.7 | 15.7 | 42.5 |
| **Global Perceived Effect ^5^** | 50 |  | 418 (-762 to 3068) | 0.05 (-0.01 to 0.10) | 9,272 | 70.9 | 29.1 | 0.0 | 0.0 |
|  |  |  |  |  |  |  |  |  |  |
| **HUMAN CAPITAL APPROACH** | | | | | | | | | |
| **Outcome** | **Sample size Outcome** | | **∆C(95%CI)** | **∆E(95%CI)** | **ICER** | **Distribution CE-plane (%)** | | | |
| **Comparison 1**  **(Int. group 1 (Usual care + TESI) vs. Control (Usual care))** | | | | | | | | | |
|  | **Int. Group 1** | **Control** | **€** | **Points** | **€/point** | **NE** | **SE** | **SW** | **NW** |
| **QALYs (0-1)^1^** | 46 | 45  45  45  45  45 | 1918 (-3142 to 6695) | 0.008 (-0.041 to 0.056) | 250,146 | 43.5 | 18.0 | 7.7 | 30.8 |
| **Back pain (0-10) ^2^** | 46 |  | 1714 (-3203 to 6585) | 0.6 (-0.5 to 1.7) | 2,772 | 11.4 | 3.9 | 24.6 | 60.1 |
| **Leg pain (0-10) ^3^** | 46 |  | 1762 (-3269 to 6577) | -0.4 (-2.2 to 1.4) | -4,497 | 53.8 | 17.0 | 10.2 | 19.0 |
| **Functioning (0-23) ^4^** | 46 |  | 1786 (-3222 to 6585) | 1.5 (-1.7 to 4.8) | 1,178 | 11.8 | 5.4 | 21.9 | 60.9 |
| **Global Perceived Effect ^5^** | 46 |  | 1675 (-3331 to 6423) | 0.04 (-0.02 to 0.11) | 39,586 | 70.5 | 24.4 | 0.5 | 4.7 |
| \| **Comparison 2**  **(Int. group 1 (Usual care + TESI) vs. Int. group 2 (Usual care + TEI))** \| \| \| \| \| \| \| \| \| \| \| \| --- \| --- \| --- \| --- \| --- \| --- \| --- \| --- \| --- \| --- \| --- \| \|  \| **Int. Group 1** \| **Int. Group 2** \| **€** \| **Points** \| **€/point** \| **NE** \| **SE** \| **SW** \| **NW** \| | | | | | | | | | |
| **QALYs (0-1)^1^** | 46 | 50  50  50  50  50 | 2331 (-2912 to 7032) | -0.012 (-0.061 to 0.038) | -200,983 | 22.2 | 8.9 | 11.4 | 57.5 |
| **Back pain (0-10) ^2^** | 46 |  | 2236 (-3049 to 7006) | -0.01 (-1.3 to 1.3) | -181,973 | 38.1 | 12.6 | 9.2 | 40.0 |
| **Leg pain (0-10) ^3^** | 46 |  | 2272 (-3021 to 7077) | -0.1 (-1.5 to 1.3) | -34,677 | 44.2 | 11.7 | 9.8 | 34.2 |
| **Functioning (0-23) ^4^** | 46 |  | 2271 (-3004 to 7101) | 0.99 (-2.15 to 4.12) | 2,281 | 19.9 | 5.5 | 15.9 | 58.8 |
| **Global Perceived Effect ^56^** | 46 |  | - | - | - | - | - | - | - |
| \| **Comparison 3**  **(Int. group 2 (Usual care + TEI) vs. Control (Usual care)** \| \| \| \| \| \| \| \| \| \| \| --- \| --- \| --- \| --- \| --- \| --- \| --- \| --- \| --- \| --- \| \|  \| **Int. Group 1** \| **Control** \| **€** \| **Points** \| **€/point** \| **NE** \| **SE** \| **SW** \| **NW** \| | | | | | | | | | |
| **QALYs (0-1)^1^** | 50 | 45  45  45  45  45 | 332 (-4466 to 5435) | 0.012 (-0.040 to 0.064) | 26,84412,019 | 37.0 | 30.8 | 13.7 | 18.4 |
| **Back pain (0-10) ^2^** | 50 |  | 403 (-4471 to 5602) | 0.8 (-0.4 to 1.9) | 518 | 6.8 | 4.9 | 37.8 | 50.5 |
| **Leg pain (0-10) ^3^** | 50 |  | 372 (-4423 to 5527) | -0.2 (-1.7 to 1.4) | -2029. | 38.0 | 25.5 | 17.6 | 18.9 |
| **Functioning (0-23) ^4^** | 50 |  | 374 (-4491 to 5642) | 0.4 (-3.3 to 4.0) | 1,045 | 24.1 | 17.3 | 27.0 | 31.6 |
| **Global Perceived Effect ^5^** | 50 |  | 343 (-4525 to 5532) | 0.05 (-0.01 to 0.10) | 7,606 | 61.8 | 38.2 | 0.0 | 0.0 |
|  |  | |  |  |  |  |  |  |  |
| **COMPLETE-CASE ANALYSIS** | | | | | | | | | |
| **Outcome** | **Sample size Outcome** | | **∆C(95%CI)** | **∆E(95%CI)** | **ICER** | **Distribution CE-plane (%)** | | | |
| **Comparison 1**  **(Int. group 1 (Usual care + TESI) vs. Control (Usual care))** | | | | | | | | | |
|  | **Int. Group 1** | **Control** | **€** | **Points** | **€/point** | **NE** | **SE** | **SW** | **NW** |
| **QALYs (0-1)^1^** | 21 | 27  27  27  27  27 | 4852 (-1989 to 12523) | -0.001 (-0.057 to 0.053) | -3,456,080 | 42.6 | 4.9 | 3.3 | 49.2 |
| **Back pain (0-10) ^2^** | 21 |  | 5046 (-2771 to 12863) | 0.8 (-0.7 to 2.2) | 6,595 | 35.9 | 1.4 | 5.0 | 57.6 |
| **Leg pain (0-10) ^3^** | 21 |  | 4854 (-3320 to 12118) | -1.7 (-3.5 to 0.1) | -2,889 | 88.0 | 6.2 | 0.3 | 5.5 |
| **Functioning (0-23) ^4^** | 21 |  | 5011 (-2725 to 12300) | -0.2 (-3.7 to 3.3) | -22,361 | 49.2 | 6.03 | 1.6 | 42.9 |
| **Global Perceived Effect ^5^** | 21 |  | 4837 (-3273 to 12186) | 0.04 (0.02 to 0.07) | 130,613 | 94.4 | 5.6 | 0.0 | 0.0 |
| \| **Comparison 2**  **(Int. group 1 (Usual care + TESI) vs. Int. group 2 (Usual care + TEI))** \| \| \| \| \| \| \| \| \| \| \| \| --- \| --- \| --- \| --- \| --- \| --- \| --- \| --- \| --- \| --- \| --- \| \|  \| **Int. Group 1** \| **Int. Group 2** \| **€** \| **Points** \| **€/point** \| **NE** \| **SE** \| **SW** \| **NW** \| | | | | | | | | | |
| **QALYs (0-1)^1^** | 21 | 25  25  25  25  25 | 364 (-6199 to 6927) | -0.044 (-0.112 to 0.023) | -8,210 | 3.9 | 7.2 | 37.4 | 51.4 |
| **Back pain (0-10) ^2^** | 21 |  | 1521 (-4837 to 7880) | 0.5 (-1.0 to 1.9) | 3,300 | 38.8 | 7.9 | 15.9 | 37.4 |
| **Leg pain (0-10) ^3^** | 21 |  | 1234 (5168 to 7616) | -0.5 (-2.4 to 1.3) | -2,247 | 59.4 | 17.3 | 8.6 | 14.7 |
| **Functioning (0-23) ^4^** | 21 |  | 963 (-5645 to 7570) | 2.0 (-2.6 to 5.6) | 488 | 7.0 | 6.5 | 31.6 | 54.8 |
| **Global Perceived Effect ^56^** | 21 |  | - | - | - | - | - | - | - |
| \| **Comparison 3**  **(Int. group 2 (Usual care + TEI) vs. Control (Usual care)** \| \| \| \| \| \| \| \| \| \| \| --- \| --- \| --- \| --- \| --- \| --- \| --- \| --- \| --- \| --- \| \|  \| **Int. Group 2** \| **Control** \| **€** \| **Points** \| **€/point** \| **NE** \| **SE** \| **SW** \| **NW** \| | | | | | | | | | |
| **QALYs (0-1)^1^** | 25 | 27  27  27  27  27 | 2840 (-3522 to 9446) | 0.026 (-0.031 to 0.083) | 108,529 | 64.1 | 16.2 | 0.8 | 18.9 |
| **Back pain (0-10) ^2^** | 25 |  | 2814 (-3573 to 9276) | 0.3 (-1.1 to 1.6) | 10,426 | 44.2 | 5.6 | 7.4 | 42.9 |
| **Leg pain (0-10) ^3^** | 25 |  | 3005 (-3423 to 9570) | 0.02 (-1.3 to 1.3) | 155,638 | 56.6 | 7.9 | 4.5 | 31.1 |
| **Functioning (0-23) ^4^** | 25 |  | 2721 (-3861 to 9218) | -1.9 (-5.7 to 1.9) | -1450 | 65.6 | 16.2 | 1.2 | 16.9 |
| **Global Perceived Effect ^5^** | 25 |  | 3031 (-3534 to 9597) | 0.04 (-0.04 to 0.11) | 81,850 | 90.5 | 9.5 | 0.0 | 0.0 |
|  |  |  |  |  |  |  |  |  |  |

C: Costs, E: Effects, ICER: Incremental Cost-Effectiveness Ratio, CE-plane: Cost-Effectiveness plane, NE: Northeast-Quadrant, SE: Southeast-Quadrant, NW; Northwest-Quadrant, ZW; Southwest-Quadrant; Cost differences were adjusted for age, gender, body mass index (BMI), and severity of back and leg pain at baseline. ^1^ adjusted for baseline utility values, work status, age, gender; ^2^ adjusted for baseline values, level of herniated disc; ^3^ adjusted for baseline values, age, gender, body mass index (BMI), and severity of back and leg pain at baseline. Lasègue's sign; ^1^ adjusted for baseline values, work status, gender; ^5^ percentage point (difference in percentage recovered between intervention -and control group) adjusted for work status. ^6^ No patients were recovered, cannot be estimated.
